# Clinical utility of SMARCA4 testing by immunohistochemistry in rare ovarian tumours

**DOI:** 10.1038/s41416-019-0687-z

**Published:** 2019-12-17

**Authors:** Catherine Genestie, Félix Blanc-Durand, Aurélie Auguste, Patricia Pautier, Ariane Dunant, Jean-Yves Scoazec, Sébastien Gouy, Philippe Morice, Enrica Bentivegna, Amandine Maulard, Audrey LeFormal, Mojgan Devouassoux-Shisheboran, Alexandra Leary

**Affiliations:** 1 Pathology Department, Gustave Roussy, Université Paris-Saclay, Villejuif, France; 2 Groupe des Investigateurs Nationaux des Cancers de l’Ovaire (GINECO)-GINECO Group for Early Phase Studies (GINEGEPS), Paris, France; 3 Gynecological Cancer Unit, Department of Medicine, Gustave Roussy, Université Paris-Saclay, Villejuif, France; 4 INSERM U981, Gustave Roussy, Université Paris-Saclay, Villejuif, France; 5 Biostatistics and Epidemiology Unit, Gustave Roussy, Villejuif, France; 6 Pathology Department, Hôpital de la Croix Rousse, Lyon, France

**Keywords:** Ovarian cancer, Diagnostic markers

## Abstract

**Background:**

Ovarian small cell carcinoma, hypercalcaemic type (SCCOHT) is a rare and lethal disease affecting young women. As histological diagnosis is challenging and urgent, there is a clear need for a robust diagnostic test. While mutations in the chromatin-remodelling gene, SMARCA4, appear to be typical, it may not be feasible routinely to be clinically relevant.

**Methods:**

Previous studies have described the value of SMARCA4 IHC to differentiate SCCOHT from ovarian neoplasms (ON), with similar histologic appearances. We aimed to evaluate its clinical utility among a cohort of 44 SCCOHT and 94 rare ON frequently misdiagnosed as SCCOHT.

**Results:**

Forty-three percent (16/36) of SCCOHT had been classified locally as non-SCCOHT confirming the diagnosis challenge. Sensitivity and specificity of SMARCA4 IHC were excellent at 88% and 94%, respectively. In a community setting with a much lower prevalence of the disease, estimated PPV is 40% while NPV remained high at 99%. Finally, among the 16 SCCOHT misclassified locally, SMARCA4 IHC testing would have resulted in corrected diagnosis in 88% of cases.

**Conclusions:**

SMARCA4 IHC is a highly sensitive, and specific test for the diagnosis of SCCOHT and is of huge clinical utility in providing a timely and accurate diagnosis of this challenging disease.

## Background

Small cell carcinoma of the ovary, hypercalcaemic type (SCCOHT) is an extremely rare, aggressive cancer affecting young women under 40 years old (median age = 24 years), associated with an average life expectancy of only 18 months.^1,2^ This disease was first described in 1975^3^ and identified as a distinct entity in 1982.^4^ Patients with SCCOHT typically present a unilateral large tumour,^4^ associated with hypercalcemia in 60% of cases^1^ and in the two-thirds of the studied cases, extraovarian spread is present.^5^ Currently, there is little consensus on the optimal management of SCCOHT, although treatment usually involves a combination of surgery, platinum-based chemotherapy and possibly radiotherapy. Recently, Witkowski et al. provided the largest and most up to date review of 293 patients with SCCOHT and collected information on stage, age and treatment modality.^2^ The most significant prognostic factors were stage and treatment, with patients undergoing high dose chemotherapy followed by autologous stem cell transplant having significantly improved survival compared to those treated with conventional post-operative chemotherapy (5 year OS 71% vs 25%, *p* = 0.002).

Morphologically, these tumours are usually composed of sheets of small closely packed cells with little cytoplasm arranged in follicle-like structures; a large cell variant has also been described.^1,6^ Despite well-established histological features, it is always challenging to distinguish SCCOHT from other rare ovarian neoplasms due to its non-specific morphology. Indeed, SCCOHT tumours can be misdiagnosed in young women as others tumours such as sex-cord stromal, germ cell, sarcoma-like (PNET, peritoneal desmoplastic round cell tumour), blastemal tumours (neuroblastoma), lymphoma, melanoma or undifferentiated epithelial ovarian tumours with obvious critical prognostic and therapeutic implications.

The “gold standard” for diagnosis remains an evaluation by an expert pathologist. In France, all suspected SCCOHT are centrally reviewed by a reference pathologist within the National Rare Ovarian Tumor Observatory (“Réseau des tumeurs malignes rares gynécologiques” http://www.ovaire-rare.org/TMRG/public/accueil_public.aspx).

However, recent publications identified alterations in the SWItch/Sucrose NonFermentable (SWI/SNF) chromatin-remodelling gene, *SMARCA4*, encoding BRG1, in SCCOHT tumours.^7–10^ In fact, among ovarian tumours, *SMARCA4* mutations are highly specific for SCCOHT tumours as mutations have been demonstrated in 85–100% of the tumours and are correlated with a complete loss of SMARCA4 protein detection by immunohistochemistry (IHC).^9,11–15^

As patients with SCCOHT frequently present in the community setting where accurate pathological diagnosis remains challenging, there is a need for a robust and accurate diagnostic assay that is feasible in routine with a rapid turn-over to avoid delays in diagnosis and treatment initiation. While S*MARCA4* mutations seems to be highly specific of SCCOHT, molecular screening for this mutation may not be feasible in routine practice.^9^ Previous studies have evaluated the sensitivity and specificity of SMARCA4 loss by IHC to differentiate SCCOHT from the more frequent epithelial ovarian cancers, mainly high grade serous histology,^11,16^ but these tumours are unlikely to be confused histologically with SCCOHT. In the same way, other teams also demonstrated that SMARCA4 loss was a useful tools to differentiate SCCOHT from histologic mimics and rare tumours of young women.^13,14^

We aimed to evaluate the clinical utility of SMARCA4 IHC testing among a large cohort of centrally reviewed SCCOHT (*N* = 44) and other rare ovarian tumours occurring in young women and frequently misdiagnosed as SCCOHT (*N* = 94). We determined the sensitivity and specificity of SMARCA4 IHC in differentiating SCCOHT from other difficult to characterise ovarian tumours that can mimic SCCOHT (sex cord, germ cell, small cell sarcomatous, blastemal and undifferentiated tumours). As the positive and negative predictive value of a diagnostic test will depend on the prevalence of the disease, we estimated its value both in a tertiary referral setting as well as in a community hospital setting. Finally, we evaluated the clinical impact of incorporating SMARCA4 IHC in the routine diagnostic algorithm by determining the proportion of patients for whom SMARCA4 IHC would have changed initial diagnosis with obvious therapeutic implications.

## Methods

### Ethics approval and consent to participate

Patients all provided written consent authorising the use of residual tumour tissue obtained during their routine diagnosis and treatment.^17^ All samples were de-identified and the specific research project was approved by the Gustave Roussy R&D committee (ref: RT12014).

### Patients selection

One hundred and thirty-eight paraffin-embedded tumours from patients referred for expert rare ovarian tumour review at Gustave Roussy Cancer Campus (GRCC) (50%) or Hospices Civils de Lyon (50%) were included. These included 44 tumours centrally confirmed as SCCOHT and a cohort of ovarian tumours mimicking SCCOHT (*N* = 94): (1) sex-cords Stromal tumours (*N* = 52) composed of juvenile (*N* = 10) and adult (*N* = 34) granulosa cells tumours, Sertoli-Leydig tumours (*N* = 4) and unclassifiable (N = 1); (2) germ cells tumours (*N* = 14) composed of embryonal carcinoma (*N* = 2), complex (*N* = 1), dysgerminoma (*N* = 4), immature teratoma (*N* = 2) and yolk sac (*N* = 5); (3) Epithelial tumours (*N* = 12) composed of undifferentiated (*N* = 10) and Neuroendocrine (NE) (*N* = 2) and finally (4) Sarcomatous and blastemal tumours (*N* = 16) composed of primitive neuroectodermal tumours (PNET) (*N* = 12), Neuroblastoma (*N* = 1), peritoneal desmoplastic round cell tumour (*N* = 1). All rare ovarian tumours are reviewed by one of the national rare tumour expert pathologists. In this study 2 of our national experts were involved. The diagnosis was made on the basis of a combination of morphological and immunohistochemical factors. Morphological features consistent with SCCOHT included: bi-phasic architecture composed of a combination of small round cells and larger cells with eosinophilic cytoplasm, rhabdoid features such as eccentric nuclei and prominent nucleoli (See sup fig S1). A comprehensive panel of IHC markers was proposed depending on morphology: after a basic panel including EMA and WT1, other markers were proposed according to cytopathological features: for undifferentiated markers such as inhibin, calretinin and SALL4, for stromal like FOXL2 and DICER1 mutations were sought. For difficult cases a further expert opinion was sought at the national ovarian rare tumour MDT.” The distribution of the diagnoses in our ‘mimickers’ cohort, is reported in Table 1.

**Table 1:** Distribution of non-SCCOHT cohort control.

**Epithelial**	
Undifferentiated carcinomas	10
Neuroendocrine carcinomas	2
Total	12 (13%)
**GCT**	
Teratoma	2
Dysgerminoma	4
Yolk sac tumour	5
Embryonal tumour	2
Complex	1
Total	14 (15%)
**Sarcoma-like and blastemal tumours**	
Desmoplastic round cell tumour	2
PNET	13
Neuroblastoma	1
Total	16 (17%)
**Sex cord**	
Adult granulosa	37
Juvenile granulosa	10
SLCT	4
Unclassified	1
Total	52 (55%)
Total	94

*PNET* primitive neuroectodermal tumour, *SLCT* Sertoli-Leydig cell tumour

### Immunohistochemistry and staining scoring

SMARCA4 (BRG1) protein expression in our cohort of SCCOHT tumours (*N* = 44) was evaluated by immunohistochemistry on whole slides. SMARCA2 (BRM) protein expression was performed in the same way on 36 tumours out of the total cohort (82%). SMARCA4 protein expression was also assessed in the non-SCCOHT cohort (*N* = 94). Assays were performed using the rabbit polyclonal antibody anti-BRG-1 (Santa Cruz, sc-10768) and anti-BRM (Abcam, ab15597), respectively, at a dilution of 1/200 and 1/50. After paraffin removal and hydration, the slides were immersed in 10 mM citrate buffer pH 6, 30 min or antigen retrieval. The antibody was incubated 1 h at room temperature, and the second antibody was incubated for 30 min at room temperature. The streptavidin labelled streptavidin-biotin amplification method (VECTASTAIN Elite ABC Kit) was carried out for 30 min followed by peroxidase/diaminobenzidine substrate/chromogen. Tumours were evaluated for nuclear staining and only those tumours showing complete absence of protein expression were classified as negative. A positive nuclear staining of lymphocytes was considered as an internal positive control. They were then scored by a pathologist, one score for staining intensity (+, ++, +++, 0) and for each intensity, the cellularity was estimated in percentage (%). The sum total of products of cellularity and intensity gave rise to a final score (H-score) for each tumour (0–300).

### Descriptive statistics diagnostic performance

Classical 2 × 2 tables were used for estimating the diagnostic performance of SMARCA4 IHC. The diagnostic performance of the SMARCA4 marker was calculated for the diagnosis of SCCOHT compared with other selected mimicking ovarian neoplasms as a whole, and then compared with other subtypes for the subtype specificity. The parameters of the test were evaluated (Sensitivity (Se), Specificity (Sp), Positive Predictive Value (PPV), Negative Predictive Value (NPV) and accuracy (A: true positives plus true negatives divided by all cases) and were reported with their 95% Confidence Interval (95% CI).

## Results

### Sensitivity and specificity of SMARCA4 IHC as a diagnostic test for SCCOHT among rare ovarian tumours

**Fig. 1:**
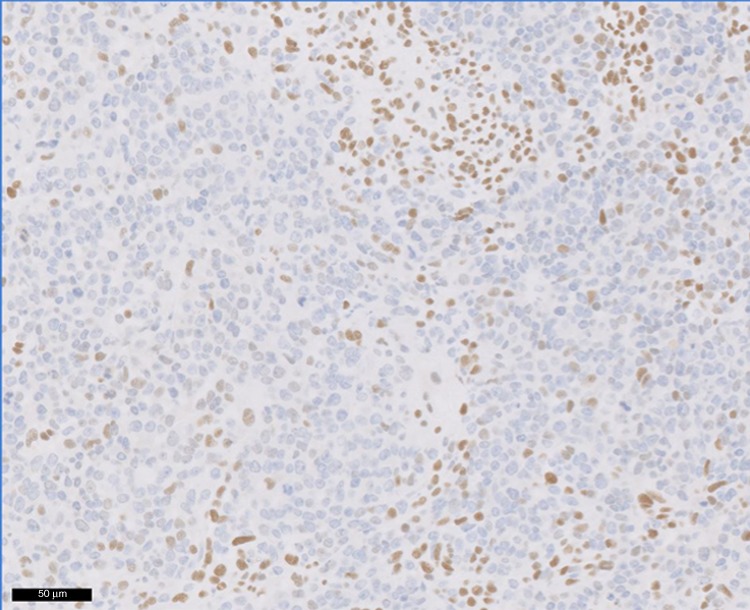
SMARCA4 expression in small cell carcinoma of the ovary, hypercalcaemic type (SCCOHT). Note positive nuclear staining of lymphocytes serving as an internal positive control.

Eighty-eight percent of SCCOHT (39/44) exhibited complete loss of SMARCA4 protein expression (Fig. 1) compared to only 6% (6/94) of mimickers (Supp Table 1) resulting in both a high sensitivity and specificity at 89% and 94%, respectively (Table 2). The six non-SCCOHT tumours that demonstrated loss of SMARCA4 were immature teratoma (*N* = 1), desmoplastic round cell tumour (*N* = 1), PNET (*N* = 1) and adult granulosa cell tumour (*N* = 3). Two of the three GCT with SMARCA4 loss were confirmed as GCT by FOXL2 mutation analysis. The third sample was old and could not generate quality DNA When considering the diagnostic performance of SMARCA4 across diagnostic classes, specificity remained homogeneous and robust at 88% for sarcomatous tumours, 92% for germ cell, 94% for sex-cord and 100% for undifferentiated epithelial ovarian tumours (Fisher exact test: *p* = 0.94) (Table 3). Positive and negative predictive values for SMARCA4 in our study were 86% and 95%, for PPV and NPV, respectively (Table 2). Importantly, the results of the IHC are unequivocal. Tumours either demonstrate complete loss (no nuclear staining) in 100% of tumour cells or significant SMARCA4 expression with a median H-score of 200 (range: 40–300, Supp Table 1). Among the 5 SCCOHT with retained expression of SMARCA4, the H-score was high (range: 50–280).

**Table 2:** Diagnostic performance of SMARCA4 test in the GRCC study.

		SCCOHT Prevalence 32%	Non-SCCOHT Prevalence 68%	
Test outcome	SMARCA4−	39	6	PPV: 86.7% (73.8%–93.7%)^a^
	SMARCA4+	5	88	NPV: 94.6% (88.0%–97.7%)^a^
		Sensitivity: 88.64% (76.2%–95.1%)^b^	Specificity: 93.6% (86.8%–97.0%)^b^	
Accuracy	92.0%			

*PPV*: positive predictive value; *NPV*: negative predictive value

^a^Standard logit Mercaldo 95% CI

^b^Wald 95% CI

**Table 3:** Specificity according to Non-SCCOHT classes of diagnoses (with Clopper- Pearson exact 95%CI).

		Total non-SCCOHT *n* = 94	Sex-cords stromal *n* = 52	Germ cell tumours *n* = 14	Epithelial *n* = 12	Sarcoma-like and blastemal tumours *n* = 16
SMARCA4 Test	Negative	6	3	1	0	2
	Positive	88	49	13	12	14
		Sp = 94% (87%–97%)^a^	Sp **=** 94% (84%–98%)^a^	Sp = 92% 67%–100%)^a^	Sp = 100% (76%–100%)^a^	Sp = 88% (64%–98%)^a^

^a^Standard logit Mercaldo 95% CI

### SMARCA4 IHC predictive value according to setting

The predictive value of a diagnostic test depends on the prevalence of the disease. In our study enriched for SCCOHT (32% of tumours analysed), both PPV and NPV were high. Since the greatest added value of this diagnostic assay is likely to be in the community setting where SCCOHT are a rare occurrence, we also modelled the predictive values in a local hospital pathology setting. We hypothesised that the prevalence of SCCOHT among difficult to characterise ovarian tumours occurring in young women in a community hospital setting would be as low as 5%. As expected with a much lower incidence of disease, the PPV decreased to 40% while the NPV remained high at 99% (Table 4).

**Table 4:** PPV and NPV according to the prevalence of SCCOHT in the target population.

	Tertiary Cancer Center (GRCC cohort)	Community setting (Projected)
Prevalence of SCCOHT	32%	5% (Estimated)
PPV	86.7% (73.8%–93.7%)^a^	40% (21%–62%)^a^
NPV	94.6% (88.0%–97.7%)^a^	99% (93%–100%)^a^

^a^Standard logit Mercaldo 95% CI

### Clinical implication of SMARCA4 IHC for diagnosis and management

All 44 tumours classified as SCCOHT were evaluated by an expert rare ovarian tumour pathologist. Among these, 36 were reviewed as a second opinion in the setting of a difficult to characterise ovarian tumour in a young patient (Supp Table 1). Initial local pathology reports were collected and for almost half (16/36 = 44%), the initial diagnosis was different thus illustrating the diagnostic challenge posed by these rare tumours. The main misdiagnosis was juvenile granulosa (6/16 = 38%) but a large variety of other histological diagnoses were suggested including desmoplastic round cells tumours, germ cell tumours, or clear cell ovarian tumours (Table 5). Importantly in many of these cases, the initial diagnosis would have resulted in a drastically different prognosis and management pathway. For example, desmoplastic round cell or epithelial ovarian tumours are managed completely differently from SCCOHT. For two of the 16 tumours mislabelled as non-SCCOHT, SMARCA4 expression was retained and thus would not have improved local diagnostic accuracy. However, for 14/16 (88%) of cases initially misdiagnosed, SMARCA4 IHC would have oriented the local pathologist towards the correct diagnosis with obvious and crucial therapeutic implications.

**Table 5:** Discordance between initial and centralised diagnosis, impact of SMARCA4 immunostaining on the change of diagnostic.

ID	Initial diagnosis	SMARCA4	Final diagnosis	Change of diagnosis
5	Carcinosarcoma or mesothelioma	**−**	SCCOHT	Yes
6	Juvenile Granulosa	**−**	SCCOHT	Yes
8	Serous carcinoma	**−**	SCCOHT	Yes
11	Desmoplastic round cells tumour	**−**	SCCOHT	Yes
13	Clear cell carcinoma	**−**	SCCOHT	Yes
14	Yolk Sac	**−**	SCCOHT	Yes
20	Seminoma	**−**	SCCOHT	Yes
21	Juvenile Granulosa	**−**	SCCOHT	Yes
23	Desmoplastic round cell tumour	+	SCCOHT	No
25	Immature Teratoma	**−**	SCCOHT	Yes
28	Juvenile Granulosa	**−**	SCCOHT	Yes
30	Papillary adenocarcinoma	+	SCCOHT	No
32	Transitional cell carcinoma	**−**	SCCOHT	Yes
34	Juvenile Granulosa	**−**	SCCOHT	Yes
41	Juvenile Granulosa	**−**	SCCOHT	Yes
42	Juvenile Granulosa	**−**	SCCOHT	Yes

### Alterations in other SNI/SNF genes in SMARCA4-wildtype SCCOHT

Given that 5/44 SCCOHT retained some degree of SMARCA4 expression, we explored whether these SMARCA4-positive tumours may demonstrate other SWI/SNF alterations, in particular, loss of the other closely related catalytic domain, SMARCA2. Among SMARCA4-positive SCCOHT, 40% (2/5) were SMARCA2 null. (Supp Table 1).

Whole-exome sequencing (WES) was performed on a single tumour with retained SMARCA4/SMARCA2 expression (ID 23, Supp data 2). The data were interrogated for SNVs within other SWI/SNF genes and for genes potentially involved in chromatin remodelling beyond SWI/SNF (list of genes in Supp data 2). This SMARCA4-WT tumour demonstrated somatic mutations in genes encoding two other subunits of the SWI/SNF remodelling complex: AT rich interactive domain 1 A (ARID1A) and AT rich interactive domain 1B (ARID1B). This tumour carried nonsense somatic mutations in *ARID1A* with putative bi-allelic inactivation (2 frameshifts 556 and 1005/p2285) and a nonsense mutation in *ARID1B* (one stop gained *R1944X*). ARID1A and ARID1B are very similar proteins with 60% homology and described as mutually exclusive paralogs involved in targeting the SWI/SNF complex to DNA.

## Discussion

Small cell carcinoma of the ovary, hypercalcaemic type (SCCOHT) is a rare tumour with an aggressive behaviour typically affecting young women and diagnosis can often be difficult to establish. SCCOHT can be confused with other rare tumours presenting as pelvic or ovarian masses in young women such as granulosa cell tumours, dysgerminomas, melanoma, lymphoma, primitive neuroectodermal or desmoplastic small round cell tumours, most of which have drastically different prognoses and therapeutic modalities.^18^ Unfortunately, patients often present with acute symptoms in the community where the initial diagnosis may be conducted by a non-expert pathologist.

Mutations in the *SMARCA4* gene are recurrent and typical of SCCOHT as they have been identified in 85–100% of SCCOHT tumours.^7–10^ Molecular screening for *SMARCA4* mutations could provide a useful tool to diagnose SCCOHT. Today, targeted sequencing is used in routine practice to identify hotspot activating mutations in *BRAF* or *EGFR* in melanoma or lung cancer, respectively.^19,20^ Unfortunately, in SCCOHT, *SMARCA4* mutations are not ‘hot-spot’ and can occur anywhere along the whole gene (ENST00000344626, 5392 bp) with different mutation types including splice site, missense and frameshift making targeted sequencing difficult in routine care.

Recent publications demonstrated the correlation between mutational status of *SMARCA4* gene and loss of its protein expression in 95% of the SCCOHT cases.^15^ In our cohort of SCCOHT (*N* = 44), 88% of the tumours demonstrated complete loss of SMARCA4.

Ramos et al. in 2014, like others, have previously analysed SMARCA4 expression in a large series of 485 ovarian tumours including mainly serous high grade ovarian tumours,^9,11,15^ but only a few focused on other neoplasms that can mimic SCCOHT.^13,14,16^ It should be noticed that, in this large cohort, four percent (15/360) of clear cell ovarian cancers presented a loss of SMARCA4 protein, which could be expected as they present frequent SWI/SNF alterations (e.g. *ARID1A* mutations in 50–60%).^21^

In order to be clinically relevant, SMARCA4 loss of expression must provide a sensitive and specific test able to differentiate SCCOHT from tumours that are considered in the differential diagnosis; only rare tumours affecting young women and mimicking SCCOHT were included. Thus, low grade serous, mucinous, or high grade serous ovarian cancers were excluded.

Within this series of rare ovarian tumours, the four diagnostic parameters (sensitivity, specificity, positive and negative predictive value) were high. Sensitivity and specificity around 90%, illustrate the excellent properties of the SMARCA4 test. Furthermore, there is no evidence of specificity varying within the non-SCCOHT diagnostic classes. While SMARCA4 testing has a PPV of 86% in a tertiary care referral centre for rare tumours, the projected PPV in a community hospital setting is lower; however, the NPV remains high at 99%, providing non-ovarian expert pathologist with a reliable tool to exclude SCCOHT.

Importantly, SMARCA4 IHC testing is binary, providing an all-or-none result rather than a continuous gradient of expression requiring optimisation of a cut-off. Tumours demonstrate either complete lack of nuclear expression or a high H-score (median 200). This is a critical feature when considering whether a novel biomarker will be robust, reproducible and easy to transfer to routine practice. Considering these results, SMARCA4 IHC should be performed, if any doubt exists, for a non-epithelial tumour in young women.

In a further effort to evaluate the clinical usefulness of SMARCA4 testing, we determined the proportion of SCCOHT patients for whom the initial local diagnosis would have been altered by a SMARCA4 test. For 14/16 (88%) initially misdiagnosed as non-SCCOHT, SMARCA4 IHC assessment would have corrected the diagnosis with crucial prognostic and therapeutic implications.

## Conclusion

Our results confirmed that loss of SMARCA4 protein could be used as a robust and quick SCCOHT differential diagnostic assay to clearly distinguish SCCOHT from histologically similar, yet difficult to diagnose pelvic tumours presenting in young women. Importantly, the IHC test is easily performed, available in the community and unlikely to be subject to inter-observer variability as the result is binary, either completely negative, or clearly positive. While a second central review by expert ovarian pathologists remains required, SMARCA4 IHC testing can improve local diagnostic accuracy, by ruling out SCCOHT in 99% of SMARCA4-positive cases and correcting the diagnosis in over 85% of otherwise misdiagnosed cases. SMARCA4 testing IHC should be proposed as part of the diagnostic algorithm for any non-epithelial ovarian tumour presenting in a young woman. Moreover, a significant proportion of SCCOHT are associated with germline SMARCA4 mutation, so accurate diagnosis has potential impact for the patient’s family as well. Finally, in the minority of SMARCA4-WT SCCOHT tumours, other SWI/SNF alterations might be relevant supporting the investigation of epigenetic or immune targeted strategies in this rare disease with a lethal prognosis

## Supplementary information

Supplementary Data

## Data Availability

Datasets generated for the current study are not publicly available considering confidentiality reasons. Anonymised data may be available from the corresponding author on justified request.
