# Supplementary material for: Clinical utility of SMARCA4 testing by immunohistochemistry in rare ovarian tumours
Source: Br J Cancer. 2019 Dec 17;122(4):564–8. doi: 10.1038/s41416-019-0687-z (PMC7028983; doi:10.1038/s41416-019-0687-z)
Supplement: Supplementary file 1 — Supplementary Data [file 41416_2019_687_MOESM1_ESM.docx]

**Supp Table 1: SMARCA4 and SMARCA2 protein expression in SCCOHT and non-SCCOHT OC.**

| ID | Type | Age | SMARCA4 | SMARCA2 | H-score |
| --- | --- | --- | --- | --- | --- |
| 1 | **SCCOHT** | **25** | **-** | **-** | **0** |
| 2 | **SCCOHT** | **37** | **-** | NA | 0 |
| 3 | **SCCOHT** | **23** | **-** | **-** | **0** |
| 4 | **SCCOHT** | **24** | **-** | **-** | **0** |
| 5 | **SCCOHT** | **36** | **-** | NA | 0 |
| 6 | **SCCOHT** | **21** | **-** | **-** | **0** |
| 7 | **SCCOHT** | **34** | **-** | **-** | **0** |
| 8 | **SCCOHT** | **29** | **-** | **-** | **0** |
| 9 | **SCCOHT** | **37** | **-** | **-** | **0** |
| 10 | **SCCOHT** | **25** | **-** | **-** | **0** |
| 11 | **SCCOHT** | **14** | **-** | **-** | **0** |
| 12 | **SCCOHT** | **24** | **+** | **+** | **100** |
| 13 | **SCCOHT** | **20** | **-** | **-** | **0** |
| 14 | **SCCOHT** | **27** | **-** | **-** | **0** |
| 15 | **SCCOHT** | **26** | **+** | **-** | NA |
| 16 | **SCCOHT** | **54** | **-** | NA | 0 |
| 17 | **SCCOHT** | **25** | **-** | **-** | **0** |
| 18 | **SCCOHT** | **31** | **-** | **-** | **0** |
| 19 | **SCCOHT** | **24** | **-** | **+** | **0** |
| 20 | **SCCOHT** | **27** | **-** | **-** | **0** |
| 21 | **SCCOHT** | **16** | **-** | **-** | **0** |
| 22 | **SCCOHT** | **25** | **-** | **-** | **0** |
| 23 | **SCCOHT** | **30** | **+** | **+** | **50** |
| 24 | **SCCOHT** | **20** | **-** | **-** | **0** |
| 25 | **SCCOHT** | **34** | **-** | NA | 0 |
| 26 | **SCCOHT** | **29** | **-** | **-** | **0** |
| 27 | **SCCOHT** | **14** | **-** | NA | 0 |
| 28 | **SCCOHT** | **40** | **-** | **-** | **0** |
| 29 | **SCCOHT** | **29** | **-** | NA | 0 |
| 30 | **SCCOHT** | **31** | **+** | **-** | **200** |
| 31 | **SCCOHT** | **28** | **-** | **-** | **0** |
| 32 | SCCOHT | 43 | **-** | **-** | **0** |
| 33 | **SCCOHT** | **35** | **-** | NA | 0 |
| 34 | **SCCOHT** | **30** | **-** | **-** | **0** |
| 35 | **SCCOHT** | **34** | **-** | **-** | **0** |
| 36 | **SCCOHT** | **31** | **-** | NA | 0 |
| 37 | **SCCOHT** | **26** | **-** | **-** | **0** |
| 38 | **SCCOHT** | **21** | **-** | **-** | **0** |
| 39 | **SCCOHT** | **37** | **+** | **+** | **280** |
| 40 | **SCCOHT** | **23** | **-** | **-** | **0** |
| 41 | **SCCOHT** | **28** | **-** | **+** | **0** |
| 42 | **SCCOHT** | **35** | **-** | - | **0** |
| 43 | **SCCOHT** | **NC** | **-** | **-** | **0** |
| 44 | **SCCOHT** | **NC** | **-** | **-** | **0** |

| ID | Type | Detail | SMARCA4 | H-score |
| --- | --- | --- | --- | --- |
| 1 | **Epithelial** | **NET** | **+** | **260** |
| 2 | **Epithelial** | **NET** | **+** | **300** |
| 3 | **Epithelial** | **Undifferenciated** | **+** | **290** |
| 4 | **Epithelial** | **Undifferenciated** | **+** | NA |
| 5 | **Epithelial** | **Undifferenciated** | **+** | **190** |
| 6 | **Epithelial** | **Undifferenciated** | **+** | **120** |
| 7 | **Epithelial** | **Undifferenciated** | **+** | **230** |
| 8 | **Epithelial** | **Undifferenciated** | **+** | **270** |
| 9 | **Epithelial** | **Undifferenciated** | **+** | **300** |
| 10 | **Epithelial** | **Undifferenciated** | **+** | **190** |
| 11 | **Epithelial** | **Undifferenciated** | **+** | **300** |
| 12 | **Epithelial** | **Undifferenciated** | **+** | **250** |
| 13 | **Germ cell tumor** | **Dysgerminoma** | **+** | **50** |
| 14 | **Germ cell tumor** | **Dysgerminoma** | **+** | **250** |
| 15 | **Germ cell tumor** | **Dysgerminoma** | **+** | **100** |
| 16 | **Germ cell tumor** | **Dysgerminoma** | **+** | **300** |
| 17 | **Germ cell tumor** | **Yolk sac** | **+** | **250** |
| 18 | **Germ cell tumor** | **Yolk sac** | **+** | **190** |
| 19 | **Germ cell tumor** | **Yolk sac** | **+** | NA |
| 20 | **Germ cell tumor** | **Yolk sac + Teratoma** | **+** | **280** |
| 21 | **Germ cell tumor** | **Yolk sac + Dysgerminoma** | **+** | **250** |
| 22 | **Germ cell tumor** | **Teratoma** | **-** | **0** |
| 23 | **Germ cell tumor** | **Teratoma** | **+** | NA |
| 24 | **Germ cell tumor** | **Embryonal carcinoma** | **+** | **160** |
| 25 | **Germ cell tumor** | **Embryonal carcinoma** | **+** | **280** |
| 26 | **Germ cell tumor** | **Complex** | **+** | **270** |
| 27 | **Sarcoma Like** | **Desmoplastic Round cell** | **-** | **0** |
| 28 | **Sarcoma Like** | **Desmoplastic Round cell** | **+** | **80** |
| 29 | **Sarcoma Like** | **PNET:Ewing** | **-** | **0** |
| 30 | **Sarcoma Like** | **PNET:Ewing** | **+** | **270** |
| 31 | **Sarcoma Like** | **PNET:Ewing** | **+** | **80** |
| 32 | **Sarcoma Like** | **PNET:Ewing** | **+** | **280** |
| 33 | **Sarcoma Like** | **PNET:Ewing** | **+** | **300** |
| 34 | **Sarcoma Like** | **PNET:Ewing** | **+** | **230** |
| 35 | **Sarcoma Like** | **PNET:Ewing** | **+** | **220** |
| 36 | **Sarcoma Like** | **PNET:Ewing** | **+** | **300** |
| 37 | **Sarcoma Like** | **PNET:Ewing** | **+** | **280** |
| 38 | **Sarcoma Like** | **PNET:Ewing** | **+** | **210** |
| 39 | **Sarcoma Like** | **PNET:Ewing** | **+** | **190** |
| 40 | **Sarcoma Like** | **PNET:Ewing** | **+** | **300** |
| 41 | **Sarcoma Like** | **PNET:Ewing** | **+** | **280** |
| 42 | **Sarcoma Like** | **Neuroblastoma** | **+** | **270** |
| 43 | **Sex chord stromal tumor** | **Adult Granulosa** | **-** | **0** |
| 44 | **Sex chord stromal tumor** | **Adult Granulosa** | **-** | **0** |
| 45 | **Sex chord stromal tumor** | **Adult Granulosa** | **-** | **0** |
| 46 | **Sex chord stromal tumor** | **Adult Granulosa** | **+** | **80** |
| 47 | **Sex chord stromal tumor** | **Adult Granulosa** | **+** | **210** |
| 48 | **Sex chord stromal tumor** | **Adult Granulosa** | **+** | **290** |
| 49 | **Sex chord stromal tumor** | **Adult Granulosa** | **+** | **300** |
| 50 | **Sex chord stromal tumor** | **Adult Granulosa** | **+** | **110** |
| 51 | **Sex chord stromal tumor** | **Adult Granulosa** | **+** | **300** |
| 52 | **Sex chord stromal tumor** | **Adult Granulosa** | **+** | **170** |
| 53 | **Sex chord stromal tumor** | **Adult Granulosa** | **+** | **180** |
| 54 | **Sex chord stromal tumor** | **Adult Granulosa** | **+** | **190** |
| 55 | **Sex chord stromal tumor** | **Adult Granulosa** | **+** | **300** |
| 56 | **Sex chord stromal tumor** | **Adult Granulosa** | **+** | **280** |
| 57 | **Sex chord stromal tumor** | **Adult Granulosa** | **+** | **300** |
| 58 | **Sex chord stromal tumor** | **Adult Granulosa** | **+** | **270** |
| 59 | **Sex chord stromal tumor** | **Adult Granulosa** | **+** | **300** |
| 60 | **Sex chord stromal tumor** | **Adult Granulosa** | **+** | **190** |
| 61 | **Sex chord stromal tumor** | **Adult Granulosa** | **+** | **160** |
| 62 | **Sex chord stromal tumor** | **Adult Granulosa** | **+** | **300** |
| 63 | **Sex chord stromal tumor** | **Adult Granulosa** | **+** | **120** |
| 64 | **Sex chord stromal tumor** | **Adult Granulosa** | **+** | **160** |
| 65 | **Sex chord stromal tumor** | **Adult Granulosa** | **+** | **200** |
| 66 | **Sex chord stromal tumor** | **Adult Granulosa** | **+** | **30** |
| 67 | **Sex chord stromal tumor** | **Adult Granulosa** | **+** | **60** |
| 68 | **Sex chord stromal tumor** | **Adult Granulosa** | **+** | **40** |
| 69 | **Sex chord stromal tumor** | **Adult Granulosa** | **+** | **120** |
| 70 | **Sex chord stromal tumor** | **Adult Granulosa** | **+** | **280** |
| 71 | **Sex chord stromal tumor** | **Adult Granulosa** | **+** | **230** |
| 72 | **Sex chord stromal tumor** | **Adult Granulosa** | **+** | **100** |
| 73 | **Sex chord stromal tumor** | **Adult Granulosa** | **+** | **300** |
| 74 | **Sex chord stromal tumor** | **Adult Granulosa** | **+** | **110** |
| 75 | **Sex chord stromal tumor** | **Adult Granulosa** | **+** | **230** |
| 76 | **sex chord stromal tumor** | **Adult Granulosa** | **+** | **60** |
| 77 | **Sex chord stromal tumor** | **Adult Granulosa** | **+** | **290** |
| 78 | **Sex chord stromal tumor** | **Adult Granulosa** | **+** | **155** |
| 79 | **Sex chord stromal tumor** | **Adult Granulosa** | **+** | **200** |
| 80 | **Sex chord stromal tumor** | **Juvenile Granulosa** | **+** | **300** |
| 81 | **Sex chord stromal tumor** | **Juvenile Granulosa** | **+** | **290** |
|  |  |  |  |  |
| 82 | **Sex chord stromal tumor** | **Juvenile Granulosa** | **+** | **300** |
| 83 | **Sex chord stromal tumor** | **Juvenile Granulosa** | **+** | **300** |
| 84 | **Sex chord stromal tumor** | **Juvenile Granulosa** | **+** | **100** |
| 85 | **Sex chord stromal tumor** | **Juvenile Granulosa** | **+** | **270** |
| 86 | **Sex chord stromal tumor** | **Juvenile Granulosa** | **+** | **190** |
| 87 | **Sex chord stromal tumor** | **Juvenile Granulosa** | **+** | **180** |
| 88 | **Sex chord stromal tumor** | **Juvenile Granulosa** | **+** | **300** |
| 89 | **Sex chord stromal tumor** | **Juvenile Granulosa** | **+** | **120** |
| 90 | **Sex chord stromal tumor** | **SLCT** | **+** | **80** |
| 91 | **Sex chord stromal tumor** | **SLCT** | **+** | **300** |
| 92 | **Sex chord stromal tumor** | **SLCT** | **+** | **160** |
| 93 | **Sex chord stromal tumor** | **SLCT** | **+** | **250** |
| 94 | **Sex chord stromal tumor** | **Unclassified** | **+** | **200** |

NA: not available

NET: neuroendocrine tumor

PNET: primitive neuroectodermal tumor

SLCT: Sertoli-Leydig cell tumor

**Supplementary Figures**

**
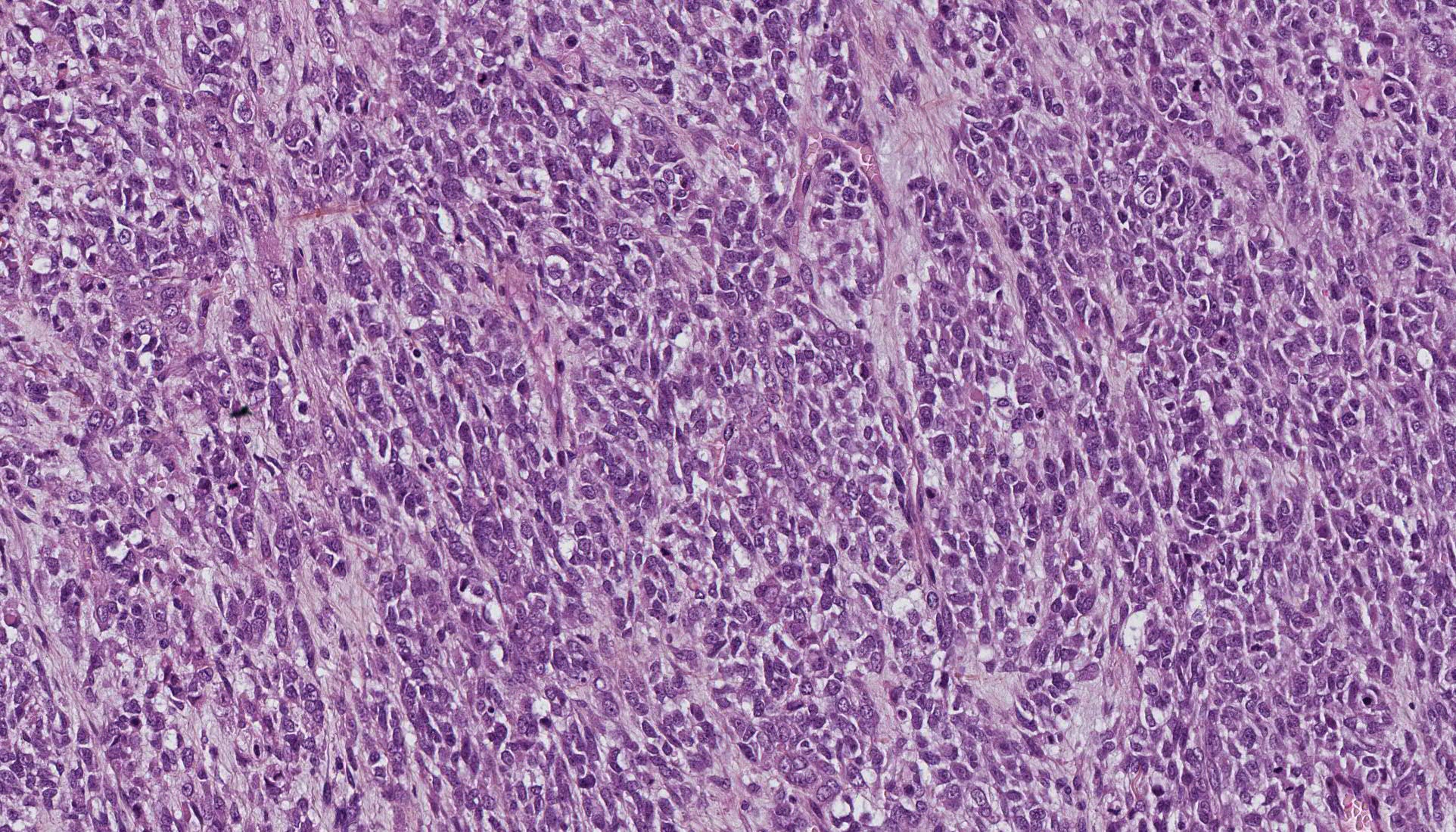

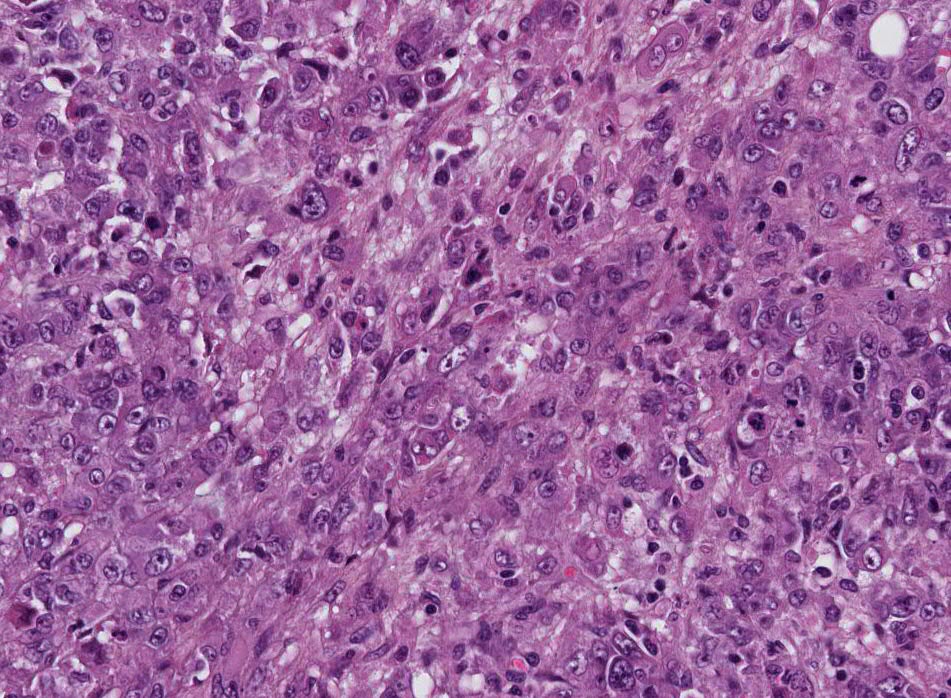
**

Figure S1 : Typical morphology in a SMARCA4+ SCCOHT (H&E). Diffuse arrangement of small basophilic cells and large eosinophilic cells with excentric nuclei (rhabdoid).

**Supplementary Data 1**

The 95%CI are estimated with the Wald method for Se and Sp and with Mercaldo's method described below for PPV and NPV [15]. The variation of specificity according to the 4 main diagnostic classes of the non SCCOHT patients was studied with an exact Fisher test. Because of small numbers in the 4 subclasses, exact Clopper Pearson 95%CI were provided. Considering published data, we hypothesized that performance of SMARCA4 marker by IHC will show 80% sensitivity and 95% specificity.

Since the main objective of this study was to evaluate the interest of the IHC SMARCA4 test for diagnosis of SCCOTH in a targeted population of young women, the PPV and NPV are the parameters of main interest. Unlike Se and Sp, the values of PPV and NPV depend on the relative prevalence of the SCCOTH/non SCCOTH diagnoses in the population where the test could be used (Mercaldo’s formula). For coherence of methodology, all the PPV and NPV 95%CI are estimated with that method. For young women with a difficult to characterize ovarian or peritoneal tumor, we hypothesized that the repartition of non-SCCOHT tumors would be a third sex cord stromal tumors, a third germ cell tumors and a third undifferentiated epithelial or sarcomatous tumors.

Two different sets of estimations are presented depending on the prevalence of SCCOTH in the targeted population: GRCC is a “Tertiary reference center for rare tumors” with a much higher prevalence of SCCOTH than in a “community Hospital” with an expected prevalence for SCCOTH of no more than 5% among difficult to characterize ovarian tumors. The analysis was performed with SAS® Version 9.3.

Statistical formula

$PPV=\frac{Se \times p}{\left( Se \times p \right) + \left( 1-Sp \right) \times(1-p)}$,

$NPV=\frac{Sp \times(1-p)}{\left( (1-Se) \times p \right) + \left( \mathrm{Sp} \right) \times(1-p)}$,

Where p denotes the prevalence of SCCOTH in the target population, PPV and NPV are reported with their 95% CI using the standard logit method of Mercaldo et al 2007.

**Supplementary Data 2**

**Whole exome sequencing (WES)**

Frozen tumors from the patient of interest was centrally reviewed for histological diagnosis and assessment of tumor cellularity, conducted by an expert pathologist. Matched blood was available for this patient. She provided written informed consent allowing use of her tumor and healthy tissues for research. Approval from the hospital’s institutional review board was obtained and funding via an educational grant awarded by the Foundation Gustave Roussy. All data were anonymized.

DNA was extracted using the [AllPrep DNA Mini Kit](http://www.qiagen.com/products/catalog/sample-technologies/rna-sample-technologies/dna-rna-protein/allprep-dnarna-mini-kit) (Qiagen, Valencia, California, USA) according to the manufacturer’s instructions. Quantity was assessed by Qubit (Life Technologies, Saint-Aubin, France). The quantity of the extracted DNA was evaluated using a Qubit spectrophotometer from Invitrogen. DNA integrity was measured using an Agilent BioAnalyzer.

Sequencing was performed on matched tumor and normal using Illumina HiSeq 2000 in pair-end mode with a mean depth of 100X producing 100bp reads. Reads were then mapped using BWA (V0.7.5a-r405) [21] with MEM algorithm against reference genome hg19. Then, analysis of coverage was done using GATK (2.7.4-g6f46d11) [22] DepthOfCoverage. Local realignment was performed using GATK around indels using GATK RealignerTargetCreator and GATK IndelRealigner.

Variants were called using Varscan2 [23], using hg19 as the reference genome requiring a minimum tumor read depth of 6, a minimum somatic read depth of 8 and a minimum allelic frequency of 0.10. Results were then annotated using SnpEff (3.3c) [24] and SnpSift (3.3c) [25] with dbSNP (v138_hg19) ([http://www.ncbi.nlm.nih.gov/SNP/](http://www.google.com/url?q=http%3A%2F%2Fwww.ncbi.nlm.nih.gov%2FSNP%2F&sa=D&sntz=1&usg=AFQjCNFDwbS2wUr9yJEQhV8uDgN6wAfdmg)) and dbNSFP (v2.1) [26]. The following further filters were applied: somatic or LOH variants; mutated allele frequency higher in tumor than normal tissue; a p-value by Fishers exact test<0.001 and variant in coding region

No *SMARCA4* or *SMARCA2* mutations were found. Similarly, no SNVs were identified within the following list of chromatin remodelling genes: *CREBBP, DNMT1, DNMT2, DNMT3A, DNMT3B, HAT1, HDAC2, HDAC3, HDAC4, HDAC7, HDAC9, HNMT3A, MYST1, MYST2, MYST4, SIN3A, SIN3B, SMARCA5, SMARCC1, ACTL6A* and *ACTL6B*.
